# A Self-Adaptive Automatic Incident Detection System for Road Surveillance Based on Deep Learning

**DOI:** 10.3390/s24061822

**Published:** 2024-03-12

**Authors:** César Bartolomé-Hornillos, Luis M. San-José-Revuelta, Javier M. Aguiar-Pérez, Carlos García-Serrada, Eduardo Vara-Pazos, Pablo Casaseca-de-la-Higuera

**Affiliations:** 1 ETSI Telecomunicación, Universidad de Valladolid, 47011 Valladolid, Spain; cbarhor@lpi.tel.uva.es (C.B.-H.); lsanjose@tel.uva.es (L.M.S.-J.-R.); javagu@tel.uva.es (J.M.A.-P.); 2 Construcciones y Obras Llorente, S.A., 47012 Valladolid, Spain; carlosgarcia@collosa.es (C.G.-S.); evara@collosa.es (E.V.-P.)

**Keywords:** automatic incident detection, smart roads, road incidents, deep learning, self-adaptivity, video surveillance, vehicle safety

## Abstract

We present an automatic road incident detector characterised by a low computational complexity for easy implementation in affordable devices, automatic adaptability to changes in scenery and road conditions, and automatic detection of the most common incidents (vehicles with abnormal speed, pedestrians or objects falling on the road, vehicles stopped on the shoulder, and detection of kamikaze vehicles). To achieve these goals, different tasks have been addressed: lane segmentation, identification of traffic directions, and elimination of unnecessary objects in the foreground. The proposed system has been tested on a collection of videos recorded in real scenarios with real traffic, including areas with different lighting. Self-adaptability (plug and play) to different scenarios has been tested using videos with significant scene changes. The achieved system can process a minimum of 80 video frames within the camera’s field of view, covering a distance of 400 m, all within a span of 12 s. This capability ensures that vehicles travelling at speeds of 120 km/h are seamlessly detected with more than enough margin. Additionally, our analysis has revealed a substantial improvement in incident detection with respect to previous approaches. Specifically, an increase in accuracy of 2–5% in automatic mode and 2–7% in semi-automatic mode. The proposed classifier module only needs 2.3 MBytes of GPU to carry out the inference, thus allowing implementation in low-cost devices.

## 1. Introduction

Cooperative-Intelligent Transport Systems (C-ITS) have deserved much attention in the last decade. The recent upraise of IoT-related projects in this field has enabled the development of systems aiming to reduce the number of fatal accidents on roads to a minimum. Specific projects in this area include the European C-ROADS [1] with many national initiatives therein such as the Spanish DGT 3.0 [2]. The deployment of these projects is organised in different scenarios or phases, one of which includes the development of automatic systems for road surveillance based on automatic video inspection. This is a widely studied field where incidents that can seriously compromise safety are detected and monitored. Acting quickly in this context can prevent secondary accidents when, for instance, a vehicle driving in the wrong way or a stopped vehicle invading the lane are detected.

Automatic Incident Detection (AID) systems based on static cameras have already been used, especially in compromised environments such as tunnels and viaducts, where compliance with safety regulations is crucial. Since manual inspection of all the acquired images is a daunting task, AID systems are becoming increasingly popular, with a certain level of automation even mandatory in some countries. Since performance of traditional systems depends on successful commissioning and calibration prior to use, there is a need for manual calibration and continuous oversight to guarantee functional performance with a minimum number of false alarms and missing incidents. This manual process can take several months under operation to enable a fully functional system. Even after that, the system needs to be frequently re-calibrated due to unforeseen circumstances, such as unexpected movements of the camera, which lead to, for instance, inaccurate detection of regions of interest in the lane. Consequently, our aim is to develop a fully automated AID system that can be easily installed with no need for human interaction for calibration. Such a system could be easily installed at a specific location in a self-adaptive, plug-and-play mode. After processing a few video frames, the system would be automatically calibrated, and all the required parameters (lane delineation, shoulder locations, driving trajectories, etc.) completely determined with no human interaction. Vehicles, pedestrians, animals, and other objects (those which might fall from a vehicle or the surrounding environment) are detected using a state of the art object detector based on deep learning. Vehicles are subsequently tracked using an artificial intelligence (AI)-based algorithm. The outputs of AI modules are then employed to detect a range of events: wrong-way drivers, stopped vehicles invading the lane, vehicles with abnormal velocities, pedestrians or animals crossing, and potentially dangerous falling objects.

The contributions of the paper are summarised as follows:

- The proposed AID system is self-adaptive and fully automated in contrast to currently operating AIDS. The attributes that event detection algorithms rely on (road segmentation and identification of driving trajectories) are automatically obtained using the output of the AI-based modules and simple algorithms. This leads to a self-calibrated system that quickly adapts to any change in the environment, thus requiring no human interaction.
- Since the event detection modules rely on object detection and tracking, these subsystems are implemented using deep learning algorithms to improve accuracy. In order to keep computational complexity low, the tracker has been notably optimised—see Section 3.2.2.
- Event detection is based on novel algorithms that have been implemented to seize all the information available from the preceding AI modules, specifically (i) existing lane delineation and shoulder detection are based on a simple yet efficient segmentation algorithm that relies on manual identification of foreground objects. A novel feature of our proposal consists of the automation of this interactive process by using the moving objects from the scene as foreground. Those objects are previously identified by a deep learning-based object detector. (ii) Driving trajectories are obtained after tracking the detected moving objects in the scene using a deep learning-based tracker. This approach is in principle more accurate than conventional methods based on optical flow, which are more sensitive to changes in light conditions, and (iii) detection of non-moving objects has been achieved by first discarding moving ones using a novel algorithm based on *inpainting* techniques [3].

The remainder of the paper is as follows: Section 2 reviews the literature in AID and Smart Roads, with specific focus on object detection and tracking and computer vision algorithms for incident detection. Section 3 describes the proposed system and modules therein. Section 4 is devoted to the validation of the system, where quantitative experimental results are presented and discussed. The metrics used for evaluation are described in Appendix A. Finally, Section 5 gathers the main conclusions obtained from this work.

## 2. Related Work: State of the Art

### 2.1. AID and Smart Roads

The term “smart” in the context of transport infrastructure extends beyond its conventional meaning of “intelligent” or “clever” to encompass “Self Monitoring Analysis and Reporting Technology” (SMART). Self-adaptive Advanced Incident Detection (AID) systems play a crucial role by facilitating three essential features of a Smart Road [4]. Firstly, these systems enable self-awareness by automatic and real-time monitoring of road conditions. Secondly, they support information interaction as intelligent devices connected to sensor networks and databases within an intelligent communication system, updating road status and triggering alarms for detected incidents. Thirdly, AID systems facilitate self-adaptation by delivering relevant information, allowing traffic to dynamically adjust to varying road circumstances.

The implementation of self-awareness relies on the processing of traffic data collected by heterogeneous AID systems, utilising a range of sensors and incident detection algorithms (e.g., inductive loops, RADARs, or cameras). Video data, rich in information, require sophisticated algorithms, including deep neural networks, for accurate incident detection, tracking, and identification of objects of interest. The flexibility and cost-effectiveness of camera installations make them the preferred choice for road monitoring, allowing efficient surveillance and incident detection through advanced image processing algorithms and decision rules.

The following subsections summarise the state-of-the-art in object detection and tracking algorithms and the specific application of computer vision for automatic incident detection.

### 2.2. Object Detection

The evolution from traditional methods relying on hand-crafted extracted features such as [5] has turned into an exclusive usage of deep learning algorithms relying on convolutional neural networks (CNN). There are two main types of CNN-based object detectors. Two-stage detectors, which firstly extract regions of interest (ROI) from the images to perform classification of these ROI in a second step. Common two-stage methods are Fast Regions with Convolutional Neural Networks (R-CNN) [6], Faster R-CNN [7], and Region-based Fully Convolutional Network (R-FCN) [8]. These methods are computationally expensive and have been replaced by one-stage detectors in real-time applications using simple platforms. Among the most commonly used one-stage detectors, the YOLO (“You Look Only Once”) series has become the most popular [9]. The YOLO architecture predicts the location of bounding boxes and classifies them in a single evaluation, similarly to the “Single Shot Detector” (SSD) [10], which also employs an end-to-end CNN for detection and classification. Additional architectures exist such as “You Look Only Twice” (YOLT) [11], which is optimised for overhead imagery with a high density of small objects.

### 2.3. Object Tracking

Further to the object detection module, a system capable of identifying detected objects uniquely throughout a sequence of video frames is required. Otherwise, the location of objects of interest could not be tracked over time to assess whether a vehicle is moving in the wrong direction from frame to frame, or a detected vehicle remains at a specific location, for instance. Object tracking is an established field, with a number of classical methods such as Mean Shift [12]. Tracking based on optical flow has also been widely used. The key idea is to obtain the relative motion of objects across different frames by estimating the velocity of those objects using space–time luminance variations [13,14,15]. Optical flow is often combined with Kalman filtering to improve accuracy. In our work, where object detection is performed prior to tracking, the use of algorithms that directly process the output of the object detector seems natural. SORT (Simple Online and Realtime Tracking) [16] uses a Kalman filter to track the bounding box parameters including an estimation of its velocity from frame to frame and their derivatives. SORT has shown high performance in tracking multiple objects in real time, which is a desirable feature for our system. An improved version of this algorithm is Deep-SORT (SORT with a Deep Association Metric) [17], which incorporates several modifications, including the use of a CNN to generate an appearance descriptor for more accurate tracking.

### 2.4. Automatic Incident Detection

AID is usually based on a series of rules established over the detected and tracked objects to decide whether a specific incident has taken part or not. Road segmentation is a first step to define such rules and needs to be achieved in an accurate an efficient manner. Image segmentation is an established field, where classical methods based on thresholding and clustering were initially overcome by more involved ones such as active contours [18] or level sets [19]. Currently, deep learning methods based on architectures such as U-Net [20] achieve higher accuracy in exchange for a reduction in efficiency. Our proposal relies on deep learning methods for object detection and tracking since accuracy at this point is of vital importance. To avoid additional efficiency drainage, we have resorted to simpler methods for road segmentation. In the AIDS proposed in [21], automation is achieved by defining a 3D map of the road where moving objects are identified from frame-to-frame pixel variations. The road is finally segmented using active contours. However, using background subtraction to identify moving objects is prone to errors. In our system, we employ the actual detected and tracked objects to improve performance, since they are available from previous modules.

To detect vehicles in the wrong direction, the normal driving direction in each lane needs to be established. This process can be manual or automatically learnt. Ref. [22] proposed a deep learning-based detector that employed YOLOv3 to detect moving vehicles, but identification of the normal driving direction is not automated. Similarly, in [23], YOLOv3 is also used, and the wrong-way direction is user-defined. In [24], the driving direction is automatically detected using the optical flow vector, though reliance on optical flow makes the system too sensitive to light conditions, as opposed to our proposal, which makes use of the actual detected moving objects. In addition, our proposal expands the dimension of the Gaussian mixture model to incorporate the position of the vehicle so as to enable detection of multiple directions in intersecting roads. Ref. [25] proposed a general-purpose system based on clustering of anomalous trajectories using support vector machines (SVMs) with Gaussian kernels. However, using SVMs for clustering increases complexity.

Methods aiming at speed estimation are usually performed in two stages, namely, perspective calibration and speed measurement. In the former, perspective is corrected so that vanishing lines become parallel after calibration. Methods such as [26] generate an homography matrix from the image vanishing points for this correction. To achieve this, lines are found using the Hough transform and edge detection, and vanishing points are sought afterwards by seeking intersection points [27]. However, there are certain cases in which this approach is not feasible due to the presence of curves in the road or the absence of clear lines in the image. To overcome this difficulty, in our work, we calculate the vanishing lines directly from the trajectories of the detected moving vehicles. Once calibration is carried out, frame-to-frame pixel displacement can be measured using optical flow vectors as in [28]. A final step transforms pixels into longitude units. This makes the final estimation difficult unless distance equivalences can be obtained from the image. Since this is not always applicable, we adopted the approach in [29], based on geolocalised points.

As for the remaining features —animal/pedestrian/object detection— most systems (e.g., [30,31]) are merely based on an object detector plus a tracker with additional rules to determine their intersection with the lanes. A recent review on this application in autonomous driving can be found in [32].

## 3. Self-Adaptive AID System for Road Surveillance

**Figure 1:**
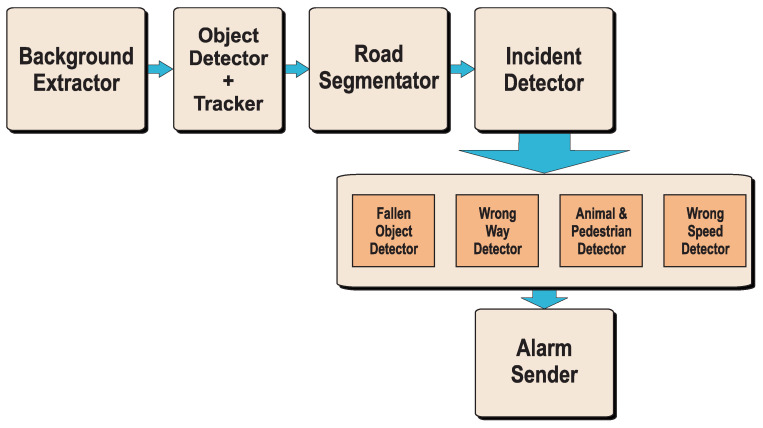
Block diagram of the proposed self-adaptive AID system.

The proposed self-adaptive system consists of several functional blocks, which are depicted in Figure 1. Self-adaptiveness, in the context of this work, consists of automatic detection of changes in the camera position leading to the need of re-segmenting the road, obtaining vehicle flow lines and directions for wrong way detection, and perspective correction for speed estimation. All this is carried out automatically without human intervention. Therefore, the cameras can be placed at different geographical locations and start operating immediately as the system will detect the new environment. Notice that this situation can also be applied when the camera is moved due to wind, for instance.

At the start of operation, the system reads a new frame from the video source and runs the background extractor, which determines whether there is a change of scene (in which case it restarts the software completely), whether there are no moving objects (in which case it reads a new frame) or whether, on the contrary, there exists object movement in the scene. Only in the latter situation, the image is processed by the detector and tracker modules, and the road is segmented. Otherwise, computations are saved to increase processing efficiency. With the road segmented and every object of interest in the frame detected and tracked, the system will detect possible incidents. Any detection triggers an alarm and sends it to a server to decide if further action is needed. Finally, the memory that is no longer being used is freed, and a new frame is read.

The following subsections describe in detail every submodule and algorithm involved in the proposed AID.

### 3.1. Background Detector

One of the first required tasks for posterior object detection is background estimation to detect sequence changes. This block uses a background subtractor based on a mixture of *K* Gaussian probability density functions (pdfs) to distinguish between the static and dynamic elements of the image. The Gaussian Mixture Model (GMM) models the colour of each pixel and is fitted using the method in [33]. The weights of the pdfs are proportional to the amount of time each colour remains on that pixel. Thus, when the weight of a pdf in a pixel is low, this pixel is classified as a foreground (dynamic) object. The main advantages of employing this background detector are detection of scene changes due to camera movements and saving of computer resources when no moving objects are detected. This allows adaptability to new conditions while keeping a low computational load and energy savings.

**Figure 2:**
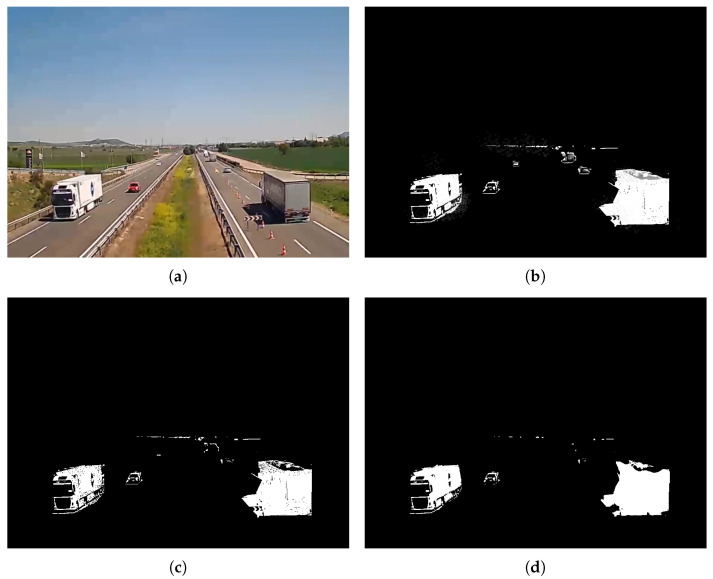
Example of background extraction using a mixture of Gaussians. (**a**) Original frame picture, (**b**) mixture of Gaussians results with shadows, (**c**) thresholding for shadow removal, and (**d**) result after filtering.

The background detector returns a mask with the pixels belonging to the background in black and the pixels corresponding to dynamic objects in white—see Figure 2. Shadows resulting from moving objects are labelled as “grey objects” within the background-foreground mask. Their identification uses a chromatic colour space to separate chromatic characteristics and illumination within foreground objects. Background and non-background pixels in the mixture are compared and shadows are detected if the differences in both chromatic and brightness components are within some thresholds.

A threshold function is thus applied to the motion mask to remove the detected shadows, and a median filter with a 5×5 kernel is applied to reduce the *salt and pepper* noise originating from slight variations in the camera angle caused, for example, by wind or small changes in lighting.

Finally, the number of white pixels (moving parts) is counted. This number is compared to an upper threshold to determine whether there is a scene change and with a lower threshold to determine whether there are significant moving objects in the image or not.

### 3.2. Object Detector and Tracker

#### 3.2.1. Object Detector

Once the background extractor has determined that there are enough moving elements, the module responsible for classifying and locating the different objects is the *detector*.

Since one of the main goals of our AID is to prioritise execution on simple devices, we have opted to implement a YOLO-type network, specifically, its fourth version, YOLOv4 [34]. This algorithm is capable of running at an acceptable speed in a medium-performance hardware, improving both speed and accuracy with respect to its previous version (v3). YOLOv3 [9] directly predicted bounding boxes and classes in a single evaluation, reaching predictions in real time. This network takes the entire image as input, divides it into cells and, for each of them, predicts a specific number of bounding boxes together with their corresponding likelihoods. In parallel, it creates a probability map for each class. Finally, Non-Maximum Suppression (NMS) [35] is applied to merge overlapping detections. Notice that, in recent years, newer versions of YOLO such as YOLOv5 [36] or YOLOv8 [37] have been proposed. These versions achieve accurate object detection while being scalable complexity-wise. As we show later, the proposed AID can work in real time with the implemented YOLOv4. Using more modern versions would result in even more fluid execution while keeping accuracy bounded and can also allow the implementation of new functionalities in the future.

The detector network was trained using thirteen classes of interest—see Table 1. For this purpose, two different datasets were used: COCO [38] and Deep-Drive [39], the latter specialised in vehicles. The first one is a big database with 896,782 images (860,001 for training and 36,781 for validation). However, only 79,009 (in the training set) and 3367 images (validation) belong to one of the 13 classes of interest. On the other hand, Deep-Drive offers 100,000 images for training and 10,000 for validation.

Since the joint dataset COCO + Deep-Drive is unbalanced, i.e., the number of images belonging to each class is very different, data balancing was initially performed. Additionally, data augmentation techniques were used to minimise overfitting, since different images are used in each epoch of the training.

Finally, it is important to note that, although the aforementioned datasets have been, and are, widely used in detection applications, both in general and in road traffic contexts, in recent years, several works have been published that focus on the development of specific datasets for incidents on roads and traffic accidents [40,41,42,43,44].

#### 3.2.2. Tracker

The tracker must meet two specifications: (i) capability of tracking several objects at the same time (multi-object) and independently of each other, and (ii) real-time execution. Following these two criteria, we decided to use a modified version of the Deep-SORT algorithm [17], which, in addition to being able of running at high speed, is sufficiently accurate in tracking multiple objects.

**Figure 3:**
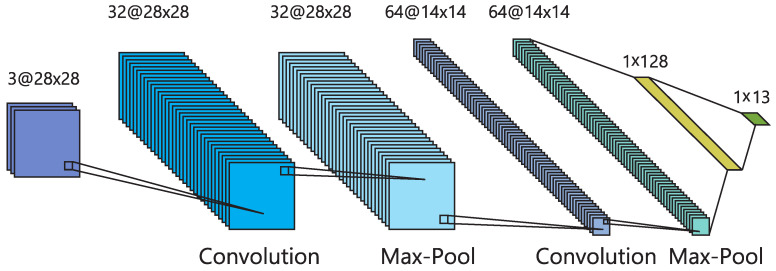
Proposed architecture of the new encoder in the object tracker.

Deep-SORT uses a deep neural network to extract high-dimensional feature embeddings from the detected objects. It employs a combination of appearance features and motion information to associate detections across frames. A Kalman filter is used for state estimation and prediction. In our work, the CNN in Deep-SORT, called *Encoder*, has been redesigned in order to reduce inference time (at the expense of a slight decrease in the feature vector quality). This way, GPU memory requirements are also reduced. Our proposal uses a CNN composed of two convolutional layers with 32 and 64 filters, respectively, using ReLU activation. Each of them is followed by a MaxPooling layer, and a fully connected layer is employed at the end of the network. Notice that, during training, an additional layer with *softmax* activation function is added so as to detect objects and to provide the probabilities of belonging to each of the 13 classes of interest. This proposed CNN architecture is shown in Figure 3.

Encoder training is performed using YOLO-detected objects, increasing their size by a 10% factor with respect to their original bounding boxes. In addition, images were resized to 28×28 pixels, and the number of objects in each class was truncated to 1294 and 71, for training and validation, respectively. In this way, the obtained classifier requires only 2.3 MBytes of GPU memory during inference. The thus obtained algorithm yields satisfactory precision, while still maintaining the ability to real-time running using low-cost devices.

### 3.3. Road Segmentation

We employed the GrabCut algorithm [45] for road segmentation, chosen for its ability to provide finer-grained selection of areas of interest within images compared to related algorithms. GrabCut is an iterative and interactive image segmentation algorithm that traditionally requires user inputs for its execution. However, we automated this manual task by incorporating a method based on identifying moving objects during the training phase. By designating the positions of these objects as foreground and creating a corresponding mask, we avoided the need for manual input. The mask remains unchanged throughout segmentation. A GMM is then utilised to classify unlabelled pixels, considering image colour statistics.

**Figure 4:**
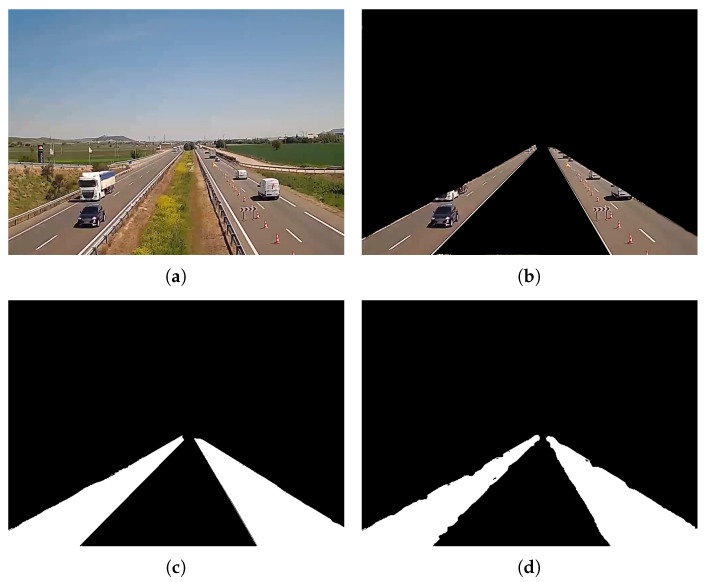
Example of road segmentation using *Grabcut* algorithm with the proposed automatic selection for foreground pixels. (**a**) Original frame, (**b**) manual segmentation, (**c**) mask of the manual segmentation, and (**d**) mask created with GrabCut.

Simulations showed good results after running the algorithm for five iterations, as can be seen in Figure 4.

### 3.4. Detection of Vehicles with Wrong Direction

In order to achieve proper detection of vehicles driving in the wrong direction (known as *kamikaze* vehicles), two different approaches have been proposed and tested.

#### 3.4.1. Detection Based on a 3D Gaussian Mixture Model

**Figure 5:**
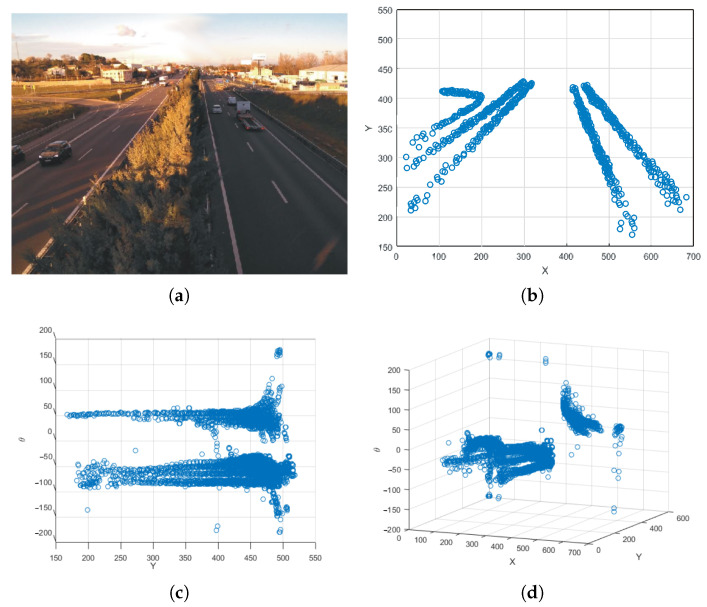
Example of data obtained during calibration phase. (**a**) Original road, (**b**) data obtained with the plan view, (**c**) data obtained with the profile view, and (**d**) data obtained with the diagonal view.

This approach requires the system to first learn the characteristics of the road by training during an initial calibration period. During this process, data necessary for all subsequent processes are collected. Unlike other systems that train for a specified number of frames [24], in our case, self-adaptation lasts until the tracker identifies a specific number of objects. This is a user configurable parameter, and a good value for it has been found to be ≈200. This way, traffic density is independent from the number of frames. During each frame of the video, the information obtained from the tracker output is collected: both the position of each vehicle and the angle of the trajectory. Figure 5 shows an example of data collected during the calibration period in a specific scenario.

In this figure, *x* represents the position of the objects along the horizontal axis of the image, *y* represents the position on the vertical axis, and θ is the angle in degrees of the path of each object. These data will be useful to (i) fit the 3D GMM that will detect vehicles in the wrong direction, (ii) perform the necessary calculations for perspective calibration used for detection of anomalous speeds (see Section 3.5), and (iii) segment the road and create an ROI that delimits the use of the algorithms to vehicles close enough to the camera. Thanks to this training, the system can automatically adapt to any environment, avoiding manual calibrations and autonomously estimate the shape of the road, the directions of the road, the average speed of vehicles, etc.

Once self-adaption finishes, kamikaze detection works as follows: the point $x={\left[x,y,\theta \right]}^{T}$ is evaluated in the pdf generated by the fitted GMM,

(1) $$\begin{array}{c} f\left(x\right)=\sum_{j=1}^{K}\omega_{j}f_{j}\left(x;\mu_{j},\Sigma_{j}\right) \end{array}$$

where f(x) is the pdf of the Gaussian mixture model, $\omega_{j}$ are the weights of the mixture, and ($\mu_{j},\Sigma_{j}$) are the mean vector and covariance matrix of each multivariate (3D) Gaussian pdf in the mixture, respectively. Parameter *K* is the number of Gaussians in the mixture, corresponding to the driving directions existing on the specific road.

The pdf value is then compared to a threshold, defined as a fraction of the weighted modes of each Gaussian using $\omega_{j}$ as weights. If f(x) is lower than the threshold value, we consider x being far enough from the distribution of the conventional traffic directions, and it is thus classified as an outlier. Every outlier is treated as a potential vehicle in the opposite direction. Finally, to confirm or reject this hypothesis, a filtering process is carried out during several frames.

#### 3.4.2. Detection Based on a Grid of Canonical Directions

This second approach is based on [24], where the image is divided using a grid, and the direction is studied in each of the cells of the grid. In contrast to [24], our work makes use of the direction vectors calculated by Deep-SORT for each of the tracked objects, instead of using optical flow vectors. This avoids duplication of direction calculations and makes the performance of the algorithm even more independent of light conditions.

**Figure 6:**
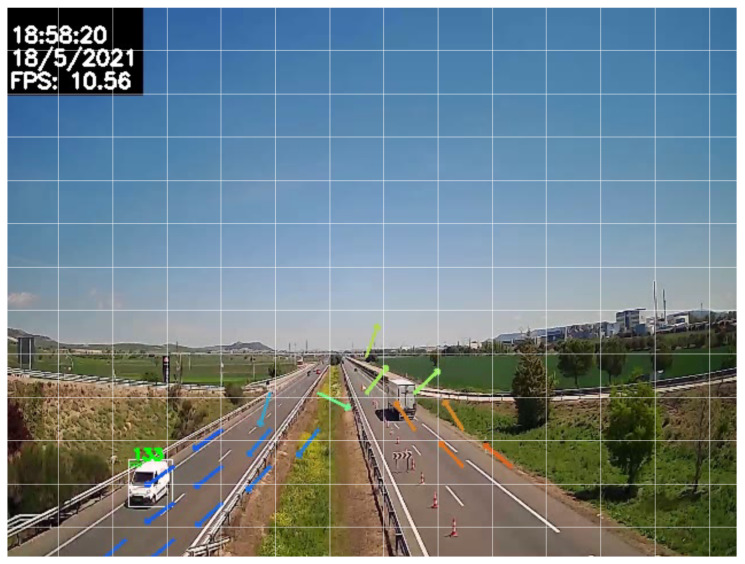
Example of wrong-way detection system using grid of canonical directions.

Specifically, a valid direction of travel is established for each cell of the grid and calculated as the moving average of the directions of each vehicle in that particular area. In order to determine whether kamikaze vehicles exist or not, the direction of each object is compared to the canonical direction of the cell, and, if it is too different, the vehicle is considered as *kamikaze*. Figure 6 shows the canonical direction vectors plotted in each of the cells of the grid.

The angle of the canonical direction vector and that of the direction vector of the vehicle under study are compared. If this difference exceeds a configurable margin (by default $45^{\circ }$), the vehicle is considered as a possible kamikaze, and this condition will be evaluated during several consecutive frames (temporal validation).

### 3.5. Abnormal Speed Detection

Abnormal speed detection has been carried out for (1) vehicles travelling at too low speed for the road—which could also be used to detect traffic jams—and (2) vehicles travelling at speed above the legal limit. Speed estimation is undertaken following two steps: perspective calibration and measurement of the speed. The former is necessary to generate a unique equivalence between the image space and the real physical space. Perspective is corrected in such a way that vanishing lines in the image become parallel. This transformation is carried out by generating the homography matrix using the vanishing points of the image [26,27].

Once perspective is corrected, displacements of vehicles between consecutive frames can be estimated, for example, by directly measuring the displacement of the object [26], or using the Kalman filter of the tracker [46], which can provide the speed estimates. Notice that it is not necessary to know actual speeds, but rather the relationship between the speeds of all the vehicles travelling on the road.

In order to obtain the homography matrix **H**, we used four points selected in the original image. The area delimited by them is transformed into a rectangular surface in the new image [47]. In our AID, we used the trajectories generated by vehicles driving on the road (typically two) to identify the main paths of the track. For this task, data collected during training is divided into four groups as a function of their path angle (θ). Then, a line is generated for each group of data by linear regression and the two trajectories that have been followed by more vehicles are considered the main paths of the track. Afterwards, the four points given by the intersection of these two lines with the edge of the area of interest (ROI) are considered. The homography matrix can be estimated by obtaining the main vanishing point from the intersection of those lines [27]. It is generated as

(2) $$\begin{array}{c} H=\left[\begin{array}{ccc} 1 & -\frac{f_{1}}{f_{2}} & 0\\ 0 & 1 & 0\\ 0 & -\frac{1}{f_{2}} & 1 \end{array}\right] \end{array}$$

where the main vanishing point is $F_{1}={\left[f_{1},f_{2},1\right]}^{T}$, and the secondary one is $F_{2}={\left[1,0,0\right]}^{T}$.

If actual speed values are needed, the approach based on the approximation proposed in [29] is used to solve the problem of the correspondence between the real physical space and the image space. This is achieved by using the actual location of the four points identified in the image. Geolocalisation involves estimation of their physical coordinates (longitude and latitude). This way, perspective transformation is carried out through the calculation, with a high degree of accuracy, of the homography matrix H.

Once the homography matrix **H** has been estimated, the transformation of speed values between the image domain and the calibrated domain is achieved with

(3) $$\begin{array}{c} \left[\begin{array}{c} X\\ Y \end{array}\right]=\left[\begin{array}{cc} \frac{h_{1,1}+C_{2,3}y}{{\left(h_{3,1}x+h_{3,2}y+1\right)}^{2}} & \frac{h_{1,2}+C_{2,3}x}{{\left(h_{3,1}x+h_{3,2}y+1\right)}^{2}}\\ \frac{h_{2,1}+C_{1,3}y}{{\left(h_{3,1}x+h_{3,2}y+1\right)}^{2}} & \frac{h_{2,2}+C_{1,3}y}{{\left(h_{3,1}x+h_{3,2}y+1\right)}^{2}} \end{array}\right]\left[\begin{array}{c} x\\ y \end{array}\right] \end{array}$$

where $C_{i,j}$ represents the minor of **H** that corresponds to $h_{i,j}$.

**Figure 7:**
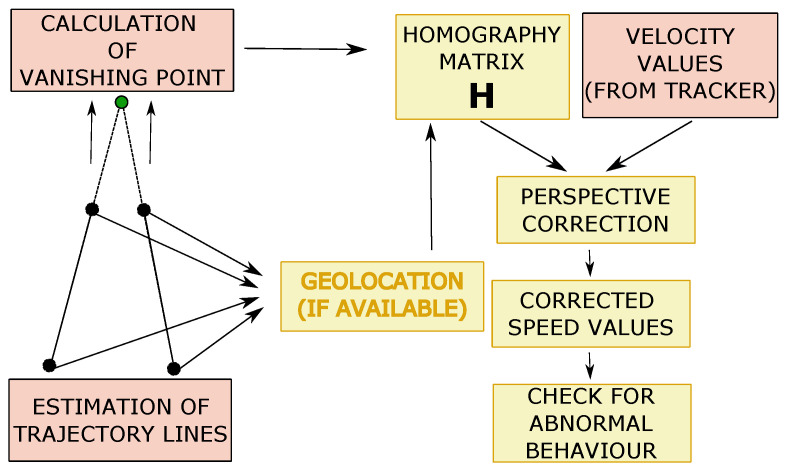
Flow chart of the overall procedure for anomalous speed detection. If geolocation is available, the homography matrix is calculated with high accuracy to provide real speed values, so that checking for abnormal values can be made using actual speed limits. Otherwise, statistics on other vehicles’ speed estimates are employed.

Therefore, speed values generated by the tracker are multiplied using Equation (3) to obtain speed in the corrected perspective domain. Afterwards, the speed of each vehicle is compared over several frames with speed thresholds. Specifically, in our case, a vehicle is classified as *too slow* if its speed is less than half of the average speed of the rest of the vehicles. On the other hand, it is classified as *too fast* if its speed is ≥10% than the average speed on that road. For the sake of clarity, Figure 7 presents a flow chart of the overall process.

### 3.6. Fallen-Object Detection

**Figure 8:**
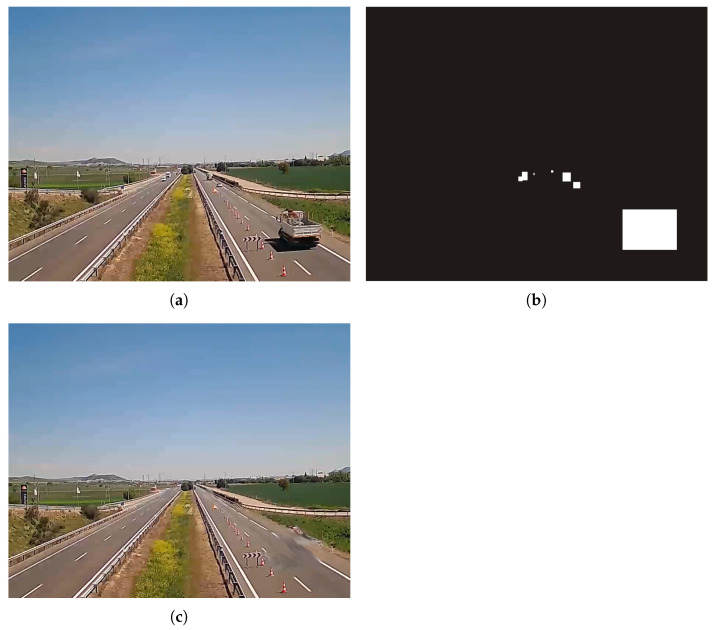
Inpainting process sample. (**a**) Original image, (**b**) generated mask for the inpainting process, and (**c**) result of the inpainting process.

Object detectors based on Deep Learning approaches need to be based on a pre-defined class set. As potentially dangerous objects in the road present a large variability in the type of objects that can appear, using object detectors can lead to poor performance unless a large database of such objects is available for training. As this was not the case, we decided to implement an alternative based on *inpainting* techniques [3]. These techniques basically consist of the removal of a specific area from the image, through the use of a mask, and the synthetic generation of a new background on that area. This way, the road without the detected vehicles can be reconstructed, and any other object in the road can be easily found by identifying changes in the sequence. We employed the method in [48] to generate a synthetic image of the road with no objects on it. This *cleaned* image acts as a template for the detection of strange objects. Specifically, objects detected by YOLO are used for generating a mask that removes every object belonging to any of the 13 classes of interest by using the inpainting process. An example of this process is shown in Figure 8.

**Figure 9:**
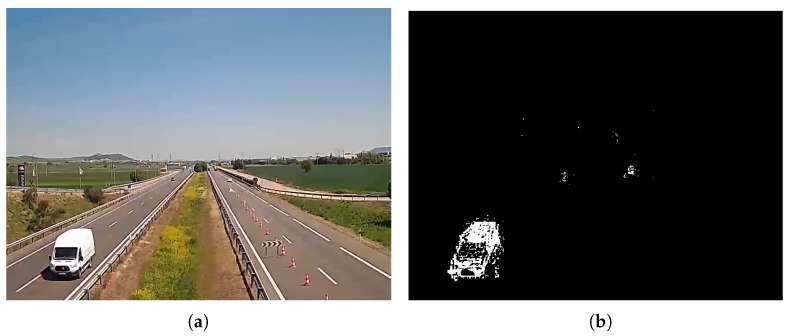
Difference image between current frame and background inpainted image. (**a**) Original image, and (**b**) difference image.

The thus obtained image is then subtracted from the current frame. This way, all objects on the road can be distinguished. The result of this process is a difference image—see Figure 9.

Finally, every detected object is filtered: objects that are not on the road and objects that overlap with the bounding boxes of the YOLO detected objects are discarded. Consequently, only those objects on the road and considered strange will be selected.

### 3.7. Animal and Pedestrian Detection

Animal and pedestrian detection is based on object detection and tracking. When YOLOv4 detects an object of class “Person” or belonging to one of the animal classes, the system evaluates whether it is on the lane for a number of frames, and, if so, it is highlighted and the corresponding alarm is triggered.

## 4. Results and Discussion

This section presents the experimental results obtained during evaluation of the proposed AID. See Appendix A for brief information on the metrics used throughout this section.

Tests were carried out using an Ubuntu Linux server with Intel Xeon^*TM*^ CPU E5-2695 v3 @ 2.30 GHz, 110 GB of RAM and graphics card nVIDIA GeForce RTX 2080 Ti, 11 GB.

Most tests were carried out using the videos shown in Table 2, where the main features of each one are shown. These videos were recorded in real roads under different meteorological, lighting, and traffic conditions.

### 4.1. Background Extractor

We paid special attention to the performance of the system in detecting scene changes as it is the main foundation of its capability to self-adapt to changing environments. This way, a test with several scene changes was ad hoc designed by concatenating sequences belonging to the eight test videos. We are interested in assessing the system performance in avoiding false positives (in this case, the system forgets everything learned and has to retrain) and false negatives (the system uses what it has learned in the previous environment instead of self-calibrating when it is actually needed). This new generated video consists of fragments of 25 frames randomly selected from the 1–8 available videos. Each video appears three times, resulting in a total of 600 frames and 23 different scene changes. Results are summarised in Table 3, showing that the system is able to identify every scene change with no false positives or negatives.

**Figure 10:**
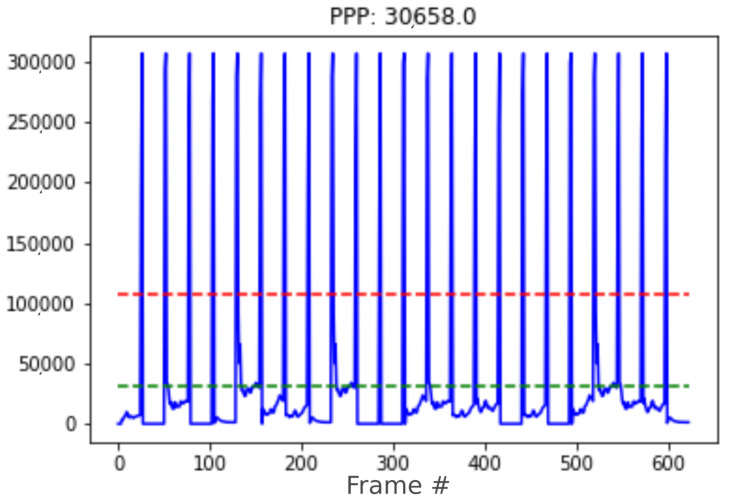
Close-up pixel plot throughout the video run. The red line dashed marks the scene change threshold. Horizontal axis: frame number.

Figure 10 shows the PPP signal (pixels in the foreground) obtained during this test. The dashed green line marks the mean value of this signal. The dashed red line marks the considered threshold value to detect a scene change.

The 23 landscape changes can be observed throughout the 600 frames. In addition, the system was tested on each of the seven videos that do not have scene changes and no false positives appeared. Finally, it was executed on video No. 6, which has a scene change, and this change was correctly identified. Therefore, we can conclude that the background extractor system for the detection of scene changes offers a satisfactory performance.

### 4.2. Object Detection

In this section, the proposed detector (YOLOv4) is tested both in its original and low-complexity (Tiny) versions. Both versions were pre-trained on the COCO dataset [38], and then transfer learning was carried out using the dataset created as explained in Section 3.2.1.

To evaluate the precision of both networks we used a completely different dataset, gathered from the MOT17det challenge, at the MOT (Multi-Object Tracking) competition [49]. This challenge has seven videos correctly labelled for the evaluation of both object detectors and trackers. These videos include a variety of sequences filmed from different viewpoints, with different lighting conditions and crowded scenarios. Table 4 shows the results obtained in the evaluation of both detectors.

The first two lines in Table 4 refer to our proposed models. It can be seen that the standard version of YOLOv4 performs considerably better than its reduced version. On the other hand, rows 3 and 4 of the table refer to the two models used, but with their original weights’ files, trained exclusively with COCO. It can be seen how our proposed training favours a more accurate detection (*mAP*) and is less prone to false negatives (higher recall) and false positives (higher precision).

### 4.3. Detection of Kamikaze Vehicles

Here, we study the alternatives described in Section 3.4. Section 4.3.1 and Section 4.3.2 present the evaluation of kamikaze detection with automatic extraction of vehicle flows and directions, which in turn reflects the capability of the system to self-adapt to changing environments. Section 4.3.3 shows results for the semi-automatic method, where vehicle directions are manually established.

#### 4.3.1. Modification of the Gaussian Mixture Model

Since having a video with enough kamikaze vehicles is unfeasible (and dangerous), we artificially modified the GMM to model just the opposite direction. This way, all vehicles appearing in the video must be identified as kamikaze vehicles. This is achieved by adding π radians phase shift to the θ data collected during training.

In this experiment, the first five videos were evaluated both with the altered GMM to account for positives (ideally all vehicles should be detected as kamikazes) as well as with the model without any change to account for negatives (ideally, all vehicles should be considered as non-anomalous vehicles). Both results are shown in Table 5.

Results in Table 5 are subject to improvement, even though they are above 0.8 in all metrics. This can be explained since real situations are always more complex than the synthetic model designed. For example, in video No. 1, the lane on the left obstructs the vision during many frames of some objects, making their detection almost impossible. The following approach notably improves these values.

#### 4.3.2. Detector Based on Grid of Canonical Directions

In order to improve previous results, the detector based on a grid of canonical directions, described in Section 3.4.2, was implemented and tested. Table 6 shows the results obtained during the execution on the same five videos.

As can be seen in Table 6, the results obtained by the automatic kamikaze detection system are promising, achieving accuracy and precision values, respectively, close to 90% and 100% in many of the evaluated scenarios. However, recall is lower than the rest of the metrics, even than the recall figure obtained with the GMM approach. This indicates that the system is more prone to false negatives than any other type of errors. Comparing these results with the GMM-based method, it can be observed that a substantial improvement has been achieved in all evaluated metrics except for recall.

Results reported in the literature can hardly be compared to our experiments. In [23,24], the systems were tested for only three and two wrong-way vehicles, respectively (simpler scenarios than ours). Successful detections were reported in all cases. Similarly, in [25], two forbidden U-turns were tested, and both of them were successfully detected. The first experiment in [22] also achieved 100% accuracy with four wrong-way drivers. Two additional experiments were carried out in this work. These are comparable to our experiments since videos were flipped to simulate vehicles flowing in the wrong direction. In these two cases, the system achieved 0.89 and 0.86 accuracies, respectively, which are slightly below our obtained results. The remaining metrics in [22] are not comparable to our results since no vehicles driving on the right direction were included in those tests.

#### 4.3.3. Semi-Automatic Detector

As false negatives are an important problem, we also considered the semi-automatic approach where direction labels for each zone are predefined, a situation that happens frequently for static cameras. For this task, we have designed an easy-to-use web application in which the operator delimits the cells of the grid and assigns the corresponding direction.

Table 7 shows the results when the semi-automatic detection system is used. This approach yields remarkable results, surpassing both the system based on a GMM and the new automatic one, in accuracy, recall, and F1. This way, this algorithm becomes a good alternative to the automatic system in complex environments with fixed cameras. However, although the automatic system is slightly worse in terms of metrics (approx. 2–3%), it offers more modularity when modelling the directions of the scenarios since each of the small cells has its own canonical address.

### 4.4. Anomalous Speed Estimation

Evaluation of the speed estimation module has been carried out using qualitative tests. This is largely due to the lack of properly labelled videos that can serve as the ground truth. Notice that the main aim is not to obtain the precision with which the system estimates the speed, but the precision the system has to identify those vehicles with an abnormally high or low speed. The two required phases are as follows.

**Figure 11:**
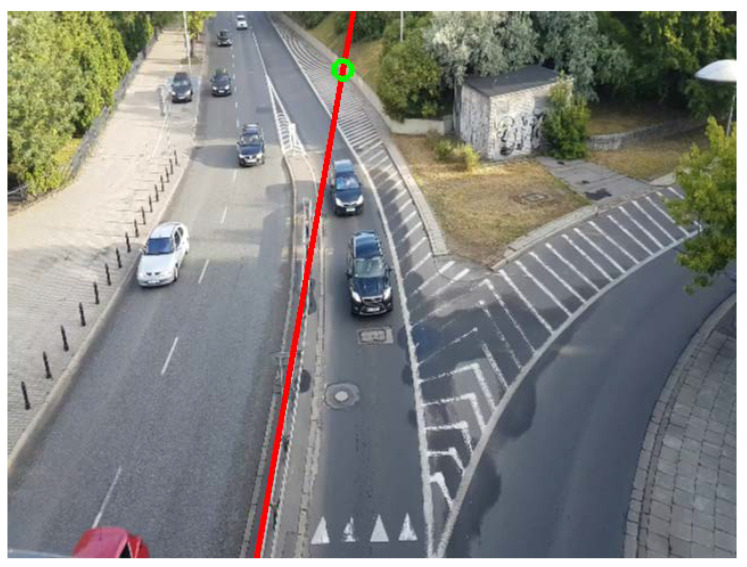
Example of path-line detection using the method in [26]. Scene No. 6.

**Figure 12:**
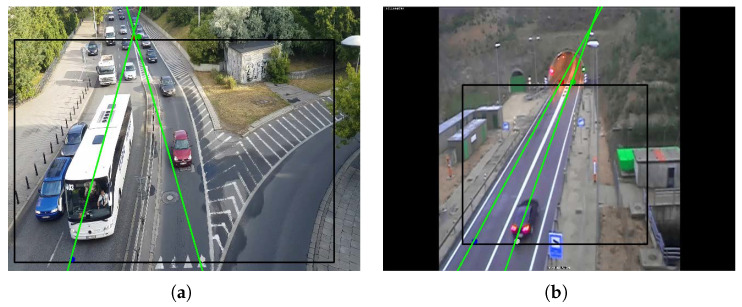
Examples of path-line detection using the proposed method. (**a**) Scene No. 6, (**b**) Scene No. 7.

**Figure 13:**
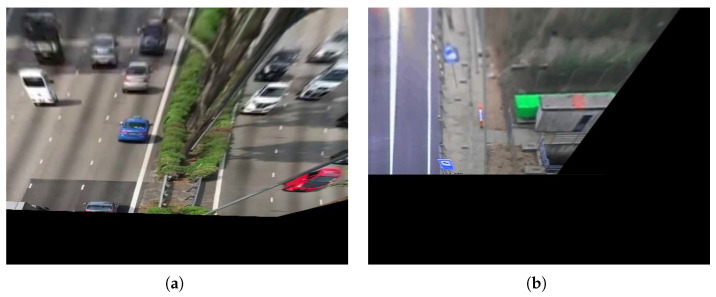
Examples of scenarios with corrected perspective. (**a**) Scenario No. 6. The perspective has been corrected for the most part. (**b**) Scenario No. 7. The perspective has been satisfactorily corrected.

**Figure 14:**
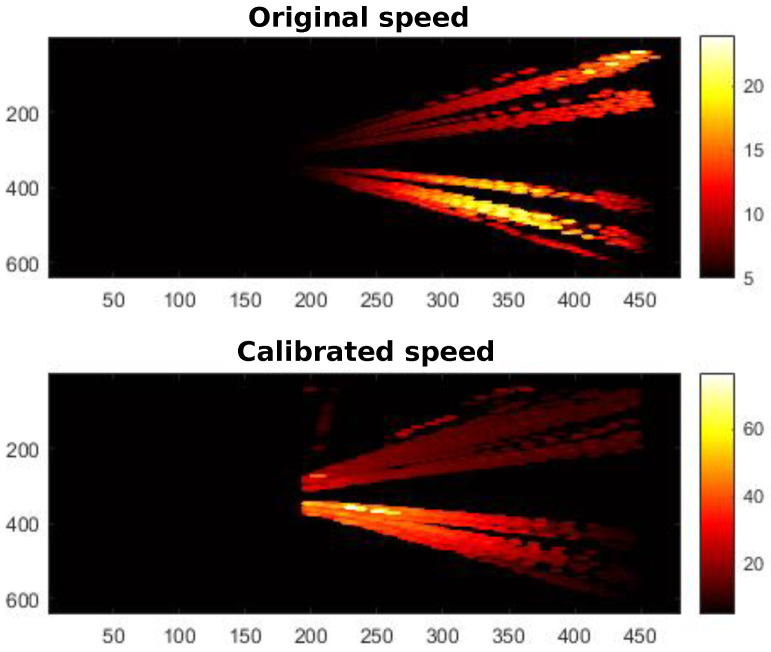
Velocity heat maps before and after perspective calibration for stage 3. Axes show position in the image.

- Perspective Calibration: First of all, to illustrate some of the drawbacks of existing approaches based on vanishing points, we show in Figure 11 an example where not enough leak lines could be detected using the Hough transform method [26], and no vanishing point could be identified.
  The Hough transform is too dependent on the characteristics of the image. The proposed method based on trajectory lines generated by the vehicles offers better results as can be seen in Figure 12, where the ROI and the intersection points with this, which will be used to find the homography matrix, are shown as well. In Figure 12b, the trajectory lines perfectly match the vanishing lines present in the image.
  After the homography matrix **H** is calculated, the image is reconstructed to check if track lanes maintain a constant width, i.e., if perspective has been corrected—see Figure 13.

- Speed estimation: Once the homography matrix **H** has been obtained, Equation (3) is used to calibrate the speed of each vehicle, previously calculated with Deep-SORT. The aim pursued is to avoid dependence of the estimated speed with respect to the position in the image. To verify these results, we used heat maps representing the speed measured before and after calibration for the different scenarios. Heat maps of speed along the road are shown in Figure 14, both before calibration (top) and after (bottom).

In Figure 14, it can be seen how the variation in the speed with position has notably decreased after calibration.

**Figure 15:**
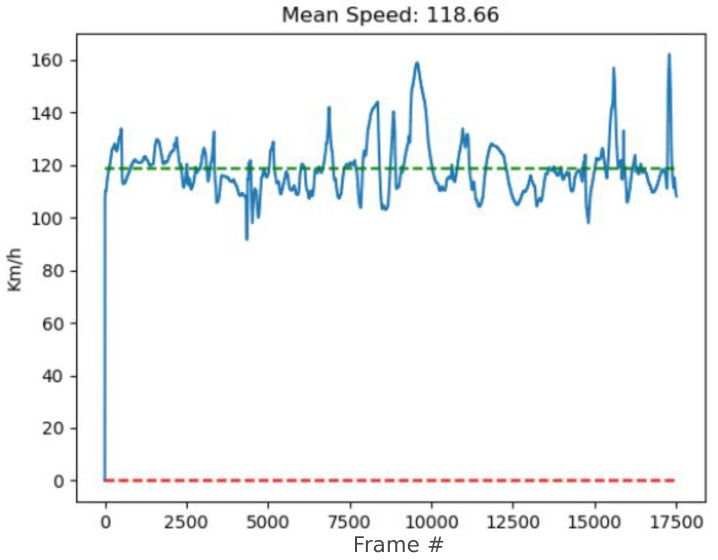
Average speed estimated in the road under study. Speed limit at this point is 120 km/h.

To assess the performance of real-speed estimation, we picked a scenario within the test videos located at coordinates 45.526646° N, 5.974535° E, corresponding to the municipality of Saint-Jeoire-Prieuré, in France. The road at this point has a speed limit of 120 km/h, so vehicles are expected to travel approximately at that speed. Figure 15 shows the variation in the average speed captured by the system throughout the entire video. As shown, the average speed is close to what was expected, sometimes showing some vertical deviation, probably caused by measurement errors in the position of vehicles in areas far from the camera.

**Figure 16:**
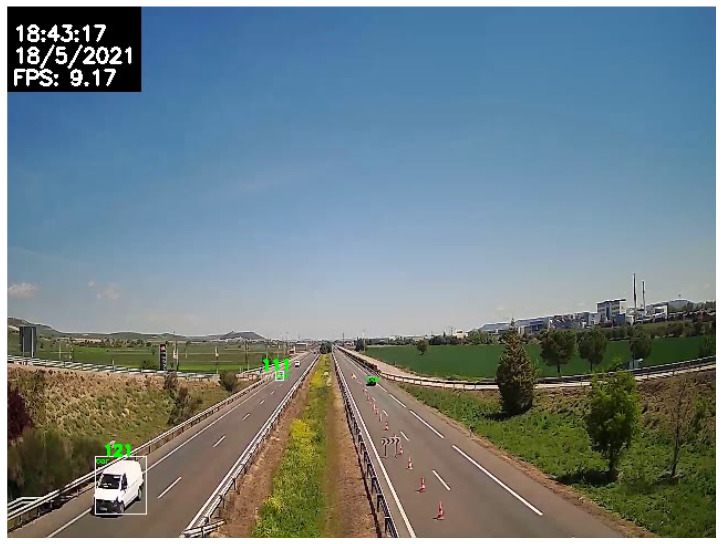
Estimation of the speed of each vehicle on the road.

Finally, Figure 16 shows the results of individual speed estimations for each vehicle. It can be seen that estimates are, in general, correct, with an average error of ∼15 km/h, according to the estimates made (standard deviation). This error is much smaller than the margin typically applied to consider a speed as abnormally high or low, which would be about 50–60 km/h up or down on a road with a baseline speed of 120 km/h. This makes the proposed system perfectly valid for our purpose.

### 4.5. Detection of Objects on the Road

We evaluated this functionality considering two aspects of the algorithm: on the one hand, the ability of the system to generate the background image, without moving objects, and on the other, the ability to detect strange objects.

It should be noted that it is not easy to find sufficiently long test videos recorded with a fixed camera, with real traffic, in which strange objects appear within a certain period of time. For this reason, a series of videos have been ad hoc recorded for this purpose and visual inspection has been chosen for testing.

**Figure 17:**
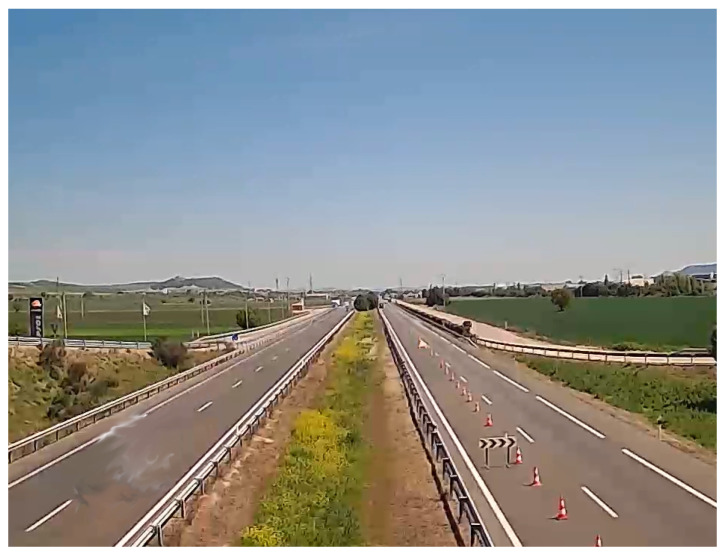
Example of mobile objects removal using the proposed inpainting technique.

**Figure 18:**
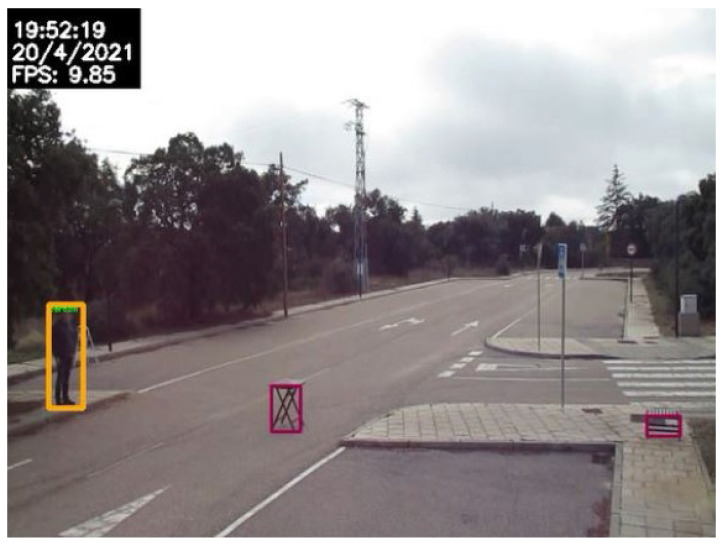
Example of fallen-object detection.

An example image obtained from the use of inpainting is shown in Figure 17. Notice that, in this case, a complex scenario has been used in which car traffic is high and at practically no time the road is seen empty. Every moving object was successfully removed. On the other hand, an example of the detection using ad hoc videos is shown in Figure 18.

### 4.6. Execution Speed and Model Complexity

This section provides results on the execution speed and complexity of the AID detection system. Execution speeds are measured in number of processed frames per second (FPS) and presented as mean ± standard deviation. Memory sizes are presented in MB and account for the size of the weights file and the GPU memory used for inference. Table 8 shows the results for the two trained YOLOv4 models.

A test similar to that proposed for the evaluation of the execution speed of the object detector has been carried out. Table 9 shows the results obtained.

As can be seen in Table 9, the original configuration [17] is two times slower than our proposed encoder. In addition, the amount of memory used with our proposal is close to 10% of the original for the weights file and 60% at the inference time.

Finally, the execution speed of all the proposed systems running in parallel was evaluated by estimating the average speed, in FPS, of the overall AID during every execution. The conclusion is that the system is capable of running at an average of 7.24 FPS, with a standard deviation of 1.65. Taking into account that only the selected tracker runs at a rate of 10.65 FPS, the loss of only 3 FPS in the execution of the rest of the systems is acceptable.

Considering now the capability of the system to detect critical incidents in real-world scenarios, a vehicle travelling at 120 km/h would take 12 s to cover 400 m, a typical field of view to be captured easily by a traffic camera. During this time, more than 80 frames would be processed, and this would be enough for our system to detect if the vehicle was travelling in the wrong direction.

## 5. Conclusions

In this work, an automatic system for detecting incidents on roads has been presented. The system is suitable for being implemented in devices with limited computational resources, since all the deep learning models within have been optimised for the sake of efficiency. In addition, the proposed system is self-adaptive and fully automatic in contrast to currently operating AIDs. Numerous tests have been carried out with different implementations of the functional modules to find the combination of algorithms providing the best performing results. Specifically, the object detector module has shown to meet expectations after specific training, both in terms of accuracy and computational complexity. In addition, a new faster encoder has been developed and trained for object tracking, being much more efficient than previous works such as [17]. Specifically, our proposed tracker processes up to twice the FPS of [17] (10.65 vs. 5 FPS), whilst requiring only 10% memory for the weights (1.2 vs. 11.2 MB) and about 40% less of memory for inference (2.3 vs. 3.83 MB).

Regarding incident detection, the performance of the automatic *kamikaze* vehicle detection module is comparable, and even better, to other works where identification of wrong-way directions is manually carried out. For instance, our method outperforms [22] by about 5–7% in detection accuracy. Furthermore, anomalous speed detection can be directly achieved after perspective correction with no need of actual speed estimation. However, the system also offers the option of estimating the speed in km/h when geolocalisation is available. Finally, an inpainting-based algorithm for detection of strange objects has been evaluated showing a 100% success rate in the removal of moving objects from the scene and no errors in the detection of static objects.

Finally, it is worth mentioning some additional advantages of the proposed AID: (i) its robustness with respect to light conditions, specially compared to those approaches based on optic flow, (ii) its capability to self-calibrate when the camera changes its position or when it suffers movements due to, for instance, wind gusts, and (iii) its additional capability to detect traffic jams by using the detection of abnormal speed functionality and counting the number of vehicles in a frame.

Future work would include testing the AID under different meteorological conditions and using more recent versions of YOLO. The main limitations of the system are given by its potential performance decrease under extreme weather conditions, especially fog and snow, where additional sensing systems would need to complement it.

## Figures and Tables

**Table 1:** Set of selected classes for YOLO detector training.

Class	Code No.	COCO	Deep-Drive
Bicycle	0	Yes	Yes
Bus	1	Yes	Yes
Car	2	Yes	Yes
Motorbike	3	Yes	Yes
Person	4	Yes	Yes
Biker	5	No	Yes
Truck	6	Yes	Yes
Cat	7	Yes	No
Dog	8	Yes	No
Horse	9	Yes	No
Sheep	10	Yes	No
Cow	11	Yes	No
Bear	12	Yes	No

**Table 2:** Summary of the features of the real recorded videos used for testing.

Video No.	One Way/Two Way	Location	Traffic Density	Characteristics
1	2	Singapore	Medium	Additional lane on the left
2	2	N/A	Medium/High	Occasional traffic jams
3	2	N/A	Medium	Aux. lane on left Lorries hiding cars
4	1	London		Curve Wind gusts
5	2	N/A	Heterogeneous High on the right	Urban Aux. lanes on both sides
6	1	N/A	Heterogeneous	Curve Aux. lane on right
7		Soria, Spain	Low	Tunnel entrance Lighting changes
8		Soria, Spain	Low	Inside a tunnel Poor lighting Glares

**Table 3:** Results obtained in the detection of scene change with the proposed background extractor.

TP	FP	TN	FN	Accuracy	Precision	Recall	F1
23	0	577	0	100%	100%	100%	100%

**Table 4:** MOT17Det results for different models of detectors.

Model	mAP	Recall	Precision
YOLOv4 (new training)	0.52	0.71	0.78
YOLOv4Tiny (new training)	0.37	0.40	0.71
YOLOv4 (COCO)	0.36	0.61	0.64
YOLOv4Tiny (COCO)	0.23	0.35	0.70

**Table 5:** Results for the automatic detection of kamikaze vehicles using the GMM.

Video No.	Accuracy	Precision	Recall	F1
1	0.84	0.83	0.86	0.84
2	0.86	0.89	0.90	0.84
3	0.83	0.82	0.84	0.83
4	0.88	0.97	0.79	0.87
5	0.86	0.92	0.78	0.85
Mean ± std. dev.	0.85±0.02	0.89±0.06	0.83±0.05	0.85±0.02

**Table 6:** Results for the automatic detection of kamikaze vehicles using grid of canonical directions.

Video No.	Accuracy	Precision	Recall	F1
1	0.90	0.96	0.82	0.89
2	0.91	0.98	0.83	0.90
3	0.90	1.00	0.80	0.89
4	0.88	0.99	0.76	0.86
5	0.87	0.99	0.75	0.85
Mean ± Std.Dev.	0.89±0.02	0.98±0.01	0.79±0.03	0.88±0.02

**Table 7:** Results for the proposed semiautomatic detection scheme of kamikaze vehicles.

Video No.	Accuracy	Precision	Recall	F1
1	0.91	0.93	0.89	0.91
2	0.87	0.96	0.78	0.86
3	0.96	0.96	0.95	0.95
4	0.92	0.94	0.89	0.91
5	0.88	0.99	0.77	0.87
Mean ± Std.Dev.	0.91±0.03	0.96±0.02	0.86±0.06	0.90±0.03

**Table 8:** Detection speed and complexity for YOLOv4 and YOLOv4 Tiny.

Model	Speed (FPS)	Memory (Weights)	Memory (Inference)
YOLOv4	61.40 ± 5.16	250	1871
YOLOv4 Tiny	110.03 ± 25.80	25	763

**Table 9:** Detection speeds and memory sizes for the tracker for different encoder options.

Model	Speed (FPS)	Memory (Weights)	Memory (Inference)
Original [17]	5.00 ± 3.4	11.2	3.83
Proposed Encoder	10.65 ± 4.56	1.2	2.3

## Data Availability

The datasets analysed during the current study are available from the corresponding author on reasonable request.

## Appendix A

This section defines the main performance metrics that have been used in this work. The classifier is evaluated using the following ones:

- True Positive (TP): The system detects something correctly.
- True Negative (TN): The system detects nothing because there is really nothing to detect.
- False Positive (FP): The system detects something when there is nothing to detect, a Type 1 error.
- False Negative (FN): The system detects nothing when there is something to detect, that is, the detection is skipped, a Type 2 error.

With these data, a confusion matrix can be constructed, where the real situation and the prediction of the system are visually confronted.

- *Precision:* Ratio of correct positive predictions to total positive predictions. Accuracy is important if false positives are to be avoided. This metric is also known as *Positive Predictive Value* (PPV).
  (A1) $$\begin{array}{c} Precision=\frac{TP}{TP+FP} \end{array}$$
- Sensitivity (*Recall*): Also called the *True Positive Rate* (TPR). It is the percentage of true positives detected versus total true positives. Sensitivity is important if false negatives are to be avoided.
  (A2) $$\begin{array}{c} Recall=\frac{TP}{TP+FN} \end{array}$$
- *Specificity*: also called the *True Negative Rate* (TNR). It is the percentage of true negatives detected versus all true negatives.
  (A3) $$\begin{array}{c} Specificity=\frac{TN}{TN+FP} \end{array}$$
- *False Positive Rate* (*FPR*): ratio of false positives to the total number of negatives.
  (A4) $$\begin{array}{c} FPR=\frac{FP}{TN+FP}=1-Specificity \end{array}$$
- *Negative Predictive Value* (*NPV*): relationship between the true negatives and the total number of predicted negatives.
  (A5) $$\begin{array}{c} NPV=\frac{TN}{FN+TN} \end{array}$$
- Accuracy: Percentage of correct predictions. However, for a dataset with different frequency of classes (unbalanced), it is not a good performance metric and others such as *PPV* and *NPV* should be used.
  (A6) $$\begin{array}{c} Accuracy=\frac{TP+TN}{TP+TN+FP+FN} \end{array}$$
- F1 score: Represents a trade-off between precision and sensitivity. It indicates how many data are classified correctly without losing a significant number of them.
  (A7) $$\begin{array}{c} F1=2\cdot \frac{precision\cdot recall}{precision+recall} \end{array}$$

The evaluation of the detection of objects within an image usually uses the following metrics:

- Intersection over the Union (IoU): compares the area and position detected with the true area and position of the object.
- Average Precision (AP): Measures the overall precision of the system taking into account all classes. The IoU metric is usually first used with a set threshold to classify the detection as true positive or false positive. Typical thresholds are IoU = 0.5, IoU = 0.75 and IoU = 0.95. Precision and sensitivity are then calculated for a single class, and their relationship is plotted. As this function is usually zigzagging, the curve is smoothed, that is, interpolated, using
  (A8) $$\begin{array}{c} p_{\mathrm{interp}}\left(r\right)=\max_{\tilde{r}\geq r}\left(p\left(\tilde{r}\right)\right) \end{array}$$
  This result is an approximate (and easier to integrate) function of the relationship between precision and sensitivity for a single class. With the interpolated curve, it is immediate to calculate the mean precision as the area under the curve.
  However, since the PASCAL VOC [50] competition, another much simpler way for the AP calculation was proposed. In this case, the value of the curve is averaged over 11 points.
  (A9) $$\begin{array}{c} AP=\int_{0}^{1}p_{\mathrm{interp}}\left(r\right)dr \end{array}$$
  (A10) $$\begin{array}{c} AP=\frac{1}{11}\sum_{r\in (0.0,0.1,\cdots ,0.9,1.0)}p_{\mathrm{interp}}\left(r\right) \end{array}$$
  With the AP calculated for each class, averaging of all the classes is performed, resulting in the mean value of the AP (*mAP*), which is widely used in object detection competitions.
